# Efficient and Safe Method for Splenic Flexure Mobilization in Laparoscopic Left Hemicolectomy: A Propensity Score–weighted Cohort Study

**DOI:** 10.1097/SLE.0000000000000884

**Published:** 2020-12-04

**Authors:** Yu-Jen Hsu, Yih-Jong Chern, Jing-Rong Jhuang, Wen-Sy Tsai, Jy-Ming Chiang, Hsin-Yuan Hung, Tzong-yun Tsai, Jeng-Fu You

**Affiliations:** * Division of Colon and Rectal Surgery, Chang Gung Memorial Hospital, Chang Gung University College of Medicine, New Taipei City; † Institute of Epidemiology and Preventive Medicine, College of Public Health, National Taiwan University, Taipei, Taiwan

**Keywords:** left hemicolectomy, standardized procedure, propensity score weighted, laparoscopic splenic flexure mobilization, minimally invasive surgery

## Abstract

Because methods of performing laparoscopic left hemicolectomy differ between surgeons, standardizing the procedure is crucial to reduce complications and secure good oncologic outcomes.

**Materials and Methods:**

This is a retrospective hospital-based cohort study using a propensity score–weighted method. This study was conducted at the department of colorectal surgery in a tertiary teaching hospital between October 2007 and December 2017. The short-term and long-term outcomes of open and laparoscopic left hemicolectomy from 10 years of experience using a standardized 4-step laparoscopic procedure at one institution were compared. Short-term outcomes were postoperative morbidity and mortality. Long-term outcomes were disease-free survival and overall survival.

**Results:**

We enrolled 564 patients who underwent open or laparoscopic left hemicolectomy for primary colon adenocarcinoma. The open surgery and laparoscopy groups had 357 and 207 patients, respectively. Compared with the open surgery group, the laparoscopy group had significantly shorter hospital stays (open vs. laparoscopy, 10 vs. 7 d, *P*<0.001), less postoperative morbidity (open vs. laparoscopy, 16.5% vs. 9.2%, *P*<0.001), and lower risks of superficial surgical site infections, lung complications, and gastrointestinal complications. No differences were observed between the groups in postoperative mortality (open vs. laparoscopy, 0.6% vs. 0.0%, *P*=0.23), disease-free survival curves (*P*=0.69), or overall survival curves (*P*=0.85).

**Conclusions:**

Our standardized 4-step technique of laparoscopic left hemicolectomy is more efficient, has fewer surgical complications, and yields better short-term and similar long-term oncologic outcomes compared with open surgery.

## Abstract 2

Supplemental Digital Content is available in the text.

Colorectal cancer (CRC) is a deadly disease with high prevalence and mortality. Most CRCs are treated surgically. Advances in surgical instruments and techniques have led to minimally invasive surgeries becoming a popular choice; in particular, laparoscopic surgery and robotic surgery are trending worldwide. Literature has reported that laparoscopic colectomy to be associated with shorter hospital stays, fewer surgical site complications, and a return to routine life.^1^,^2^ Moreover, 4 randomized trials comparing the long-term outcomes of open and laparoscopic surgery groups have shown no differences after 3-, 5-, or 10-year analyses.^3^–^7^ These studies have included different operations, namely, right hemicolectomy, left hemicolectomy, anterior resection, and total mesorectal excision. However, tumors located in the descending colon, splenic flexure, or distal transverse colon account for only 7.5% to 10% of all colon cancers.^8^,^9^ Only few studies with small sample sizes have demonstrated better short-term outcomes and similar long-term outcomes of left hemicolectomy performed laparoscopically than open.^10^–^12^

Left hemicolectomy is more challenging than other colectomies, takes longer than right hemicolectomy, and results in more superficial surgical site infections.^13^ Iatrogenic splenic injury is a major adverse event that can occur during splenic flexure mobilization surgery. It increases the risk of mortality and morbidity, prolongs operative time and hospital stay, and increases health care costs.^14^ Because the number of patients requiring this surgery is limited and the experience and technique of surgeons differ, standardizing the surgical procedure of laparoscopic left hemicolectomy is crucial to prevent complications and secure good oncologic outcomes. Moreover, propensity scores estimated through weighting are preferable for analyzing time-to-event outcomes and increasing statistical efficiency with limited sample size.

Although numerous reports outline the procedure of laparoscopic left hemicolectomy, the steps vary in each. In 2007, our institution started to follow a standardized 4-step procedure for left hemicolectomy. In this study, we compared 10 years of short-term and long-term survival outcomes of open surgery and laparoscopic left hemicolectomy using our standardized laparoscopic procedure and propensity score–weighted (PSW) analysis.

## MATERIALS AND METHODS

We retrieved data regarding our clinicopathologic variables from the Colorectal Section Tumor Registry of Chang Gung Memorial Hospital (CGMH). The Institutional Review Board of CGMH approved this study (IRB No. 202000644B0).

### Patient Selection and Surgical Method

Between October 2007 and December 2017, 564 patients underwent left hemicolectomy for primary colon adenocarcinoma with tumors localized in the distal transverse colon, splenic flexure, or proximal descending colon. Preoperatively, the patients were involved weekly with a multidisciplinary team to determine the clinical cancer stage according to preoperative workup, which mainly included colonoscopy and whole-body computed tomography. The decision of surgery type, open or laparoscopic, was based on a discussion between the patient and colorectal surgeon.

### Standardized 4-step Technique for Laparoscopic Left Hemicolectomy (Video, Supplemental Digital Content 1, http://links.lww.com/SLE/A258)

Patients are placed in a supine or Lloyd-Davies position with the surgeon and the assistant surgeon, who holds the camera, standing on the patient’s right side. Pneumoperitoneum is achieved using the Veress needle method at the umbilicus, and the abdomen is insufflated with carbon dioxide gas to a pressure of 12 mm Hg. The standard technique for performing laparoscopic left hemicolectomy is to use 4 ports. An 11 mm trocar is placed around the umbilicus as the camera port, and a 12 mm trocar is inserted in the right lower quadrant on the right midaxillary line to serve as the chief working port for the surgeon. One 5 mm trocar is used in the right upper quadrant to serve as the left working port for the surgeon, and one 5 mm trocar is placed in the left lower quadrant for the assistant surgeon

#### Step 1: Ligation of the Vessel’s Pedicle: Inferior Mesenteric Vein (IMV)

**FIGURE 1:**
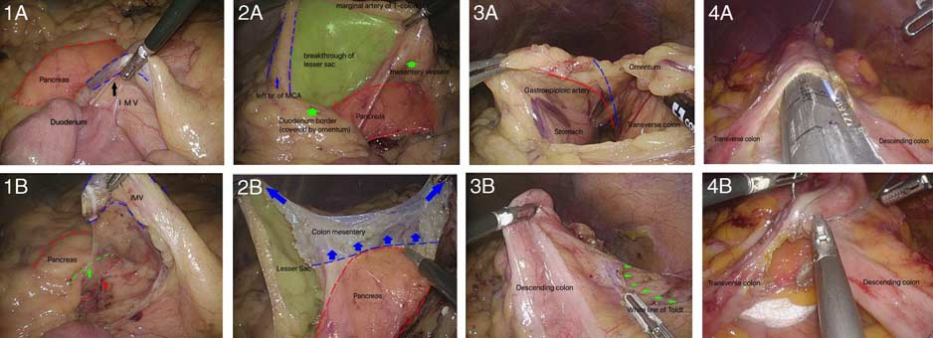
Standardized 4-step technique for laparoscopic left hemicolectomy. Step 1: Ligation of vessel pedicle: inferior mesenteric vein (IMV). By ligating and separating the IMV (1A), we can lift the mesocolon from pancreas anteriorly (green arrows). The red arrow is the common mistake to disorient to the posterior pancreas (1B). Step 2: Retroperitoneal dissection. Entering the lesser sac by lifting the left branch of middle colic artery (MCA) and vessels of mesentery (2A). By holding the mesentery near the lesser sac and near the cutting edge of the IMV to imitate a frame a canopy tent (2B), the pancreas can be easily recognized and carefully separated from the mesentery in the medial-to-lateral direction. Step 3: Mobilization of lateral attachment of bowel. First, the omentum attached to the colon is divided according to the lower border (blue line) of gastroepiploic artery (red line) (3A). Second, the lateral attachment of the descending colon along the white line of the Toldt fascia is released (3B). Step 4: Bowel anastomosis. This picture showed intracorporeal anastomosis by GIA stapler (4A) and suturing are used to close the resulting gap (4B).

A grasp is used to outstretch and raise the mesentery of the descending colon, and dissection begins at the dimpling surface of the mesentery below the IMV. The dissection plane runs along the IMV away from the retroperitoneum and toward the root of the IMV and Treitz ligament. Through this technique, the IMV can generally be clearly defined and divided (Fig. 1—1).

#### Step 2: Retroperitoneal Dissection

**FIGURE 2:**
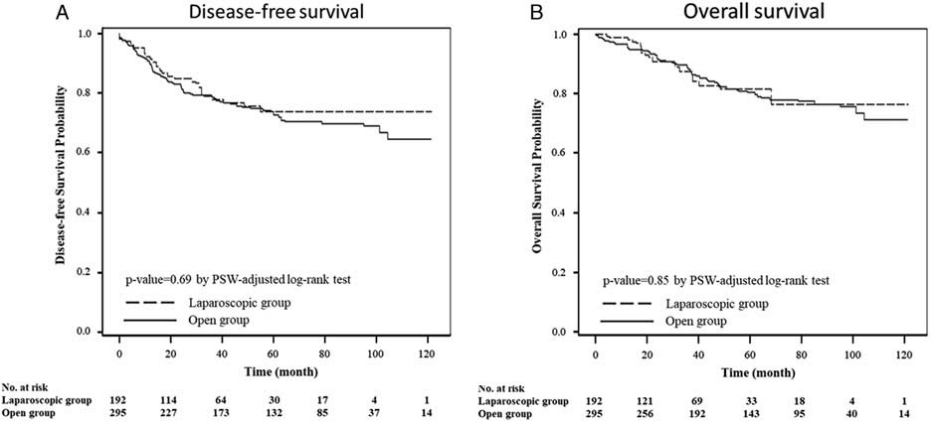
Propensity score–weighted (PSW) survival.

Retroperitoneal dissection is principally performed medial to lateral. A useful approach for separating the retroperitoneal pancreas is to identify the hotspot and enter the lesser sac, which is demarcated by the superior marginal artery of the transverse colon, inferior to the duodenum and mesenteric vessels on both sides. After the lesser sac is entered, the landmark of retroperitoneal dissection is the pancreas. By holding the mesentery near the lesser sac and near the cutting edge of the IMV to imitate a frame a canopy tent (FACT), the pancreas can be easily recognized and carefully separated from the mesentery in the medial-to-lateral direction toward the border between the splenic flexure and descending colon. The mesentery is dissected laterally by dividing the Toldt fascia above the Gerota fascia as far as possible to the descending colon (Fig. 1—2).

#### Step 3: Mobilization of Lateral Attachment of the Bowel

First, the omentum attached to the colon is divided, dissecting the gastrocolic and splenocolic ligaments to release the transverse colon and splenic flexure. Second, the lateral attachment of the descending colon along the white line of the Toldt fascia is released. Third, dissection continues along the white line of the Toldt fascia to the lateral attachment of the sigmoid colon to achieve mobilization and prevent tension during anastomosis (Fig. 1—3).

#### Step 4: Bowel Anastomosis

After complete division of the mesentery of the resected bowel, including the left colic artery, left branch of the middle colic artery, and marginal artery, colocolonic anastomosis is performed either through extracorporeal anastomosis or intracorporeal anastomosis. For extracorporeal anastomosis, the left-sided colon is exteriorized through the midline incision by extending the umbilical port wound and subsequently either stapled side to side or handsewn end to end. For intracorporeal anastomosis, both ends of the transverse and descending colons are divided using GIA staplers, and side-to-side stapler anastomosis and suturing are used to close the resulting gap. Specimens are extracted mainly by extending the umbilical wound or via a Pfannenstiel incision (Fig. 1—4).

### Covariates and Outcome Measures

We included patient variables such as age, sex, and body mass index (BMI) and health information, such as history of hypertension, cardiac disease, cerebrovascular disease, asthma, diabetes mellitus, liver cirrhosis, and previous surgery. Preoperative blood data, including carcinoembryonic antigen (CEA) (≥5 or <5 ng/mL), hemoglobin (<10 or ≥10 g/dL), and albumin (<3.5 or ≥3.5 g/dL), were recorded. Tumor size (<4 or ≥4 cm), histologic subtype (adenocarcinoma, mucinous type, signet ring cell, or undifferentiated), histologic grade (well, moderate, or poor differentiation), tumor invasion depth (T stage), and sampled and positive lymph node numbers (N stage) constituted the tumor-related variables.

We measured short-term and long-term outcomes. Short-term outcomes were postoperative morbidity and mortality, which were respectively defined as surgical complications and as death occurring within 30 days after surgery. Surgical complications included wound-related (infection or dehiscence), pulmonary (atelectasis or pneumonia), cardiovascular (myocardial infarction, stroke, or embolism), urinary tract (urinary tract infection or neurogenic bladder), gastrointestinal (obstruction, ileus, or bleeding), abdominal (abscess or internal bleeding), anastomosis (leakage or stenosis), and other rare complications.

Long-term outcomes were disease-free survival (DFS) and overall survival (OS). DFS was defined as the duration between the date of initial surgery and that of confirmation of recurrence or death from any cause. OS was defined as the duration between the date of initial surgery and date of death from any cause. Other clinical covariates, including underlying medical illnesses and preoperative laboratory values, were also compared between the groups.

### Statistical Analysis

Differing baseline characteristics of the groups to be compared can lead to confounding bias and hinder causal inference in nonrandomized studies. In such cases, propensity scores,^15^ defined as the probability of treatment assignment conditional on the measured baseline covariates, can be used to minimize such differences. Propensity scores are estimated using logistic regression. We obtained an unbiased estimate of the average treatment effect, considering the effect of whether laparoscopy could be applied, for all patients in the study by adjusting only for the estimated propensity score rather than several confounders.^15^

Propensity scores can be estimated through matching, weighting, stratification, or regression.^16^,^17^ Among these methods, matching and weighting are preferable to analyze time-to-event outcomes because they can estimate hazard ratios (HRs) with minimal bias.^18^ In our study, we applied PSW because matching may reduce statistical efficiency. PSW generates a pseudo-population in which treatment assignment is independent of measured baseline covariates. The weights for each study subject were calculated using the formula: $w\;=\;\frac{z}{e}\;+\;\frac{1\;-\;z}{1\;-\;e},$ where *z*=1 to denote the laparoscopy group, *z*=0 to denote the open surgery group, and *e* denotes the estimated propensity score. The pseudo-population method minimizes systematic differences among the groups (as in a randomized study), consequently allowing for stronger causal inference.

Categorical clinicopathologic variables, presented as frequencies and proportions, were compared using the χ^2^ and Fisher exact tests. The Wilcoxon rank-sum test was used to analyze continuous variables, expressed as medians and ranges. Balance diagnostics were used for comparing the distribution of baseline covariates of the 2 groups in the pseudo-population with *P*-values or standardized differences (SDs).^16^,^17^ The Kaplan-Meier method and a PSW-adjusted log-rank test were used to estimate and compare DFS and OS curves.^19^ Crude and PSW-adjusted HRs were obtained using Cox proportional-hazards regression models. Statistical significance was set at *P*-value<0.05 or SD>0.2. All the statistical analyses were performed using SAS 9.4 (SAS Institute, Cary, NC).

## RESULTS

In total, we enrolled and analyzed the data of 564 patients with colon cancer who underwent radical left hemicolectomy, and we divided them into the open group (357 patients) and laparoscopy group (207 patients). The mean age and median follow-up time of these patients were 63.9 years and 37.3 months, respectively.

Table 1 shows the baseline characteristics of both groups, before and after PSW. The distributions of sex, number of people with BMI > 25, history of colorectal surgery, preoperative serum CEA levels, serum albumin levels, and pathologic results (TNM stage, histologic type, histology grade, and tumor size) were significantly different between the 2 groups (*P*≤0.05, SD≥0.20) before PSW; however, this was rectified through PSW. Moreover, no significant differences were observed between the groups in other potential confounders, including proportion of people older than 65 years, history of other abdominal surgery, presence of comorbidities, hemoglobin levels, and number of retrieved lymph nodes. The mean nodal harvest in the open group was more than the laparoscopic group (29.0±16.8 vs. 26.2±12.4, respectively, *P*=0.012). There was no difference between open and laparoscopic approaches (*P*=0.91) in the ratio of lymph nodes harvested more or less than 12 after PSW.

**TABLE 1:** Baseline Characteristics of Patients Treated With Open Surgery Versus Laparoscopic Surgery, Before and After Propensity Score Weighting

	n (%)		*P*	
	Open Group (N=357)	Laparoscopic Group (N=207)	Before	After
Age (y)			0.33	0.96
<65	186 (52.10)	99 (47.83)		
≥65	171 (47.90)	108 (52.17)		
Sex			0.05	0.76
Female	183 (51.26)	88 (42.51)		
Male	174 (48.74)	119 (57.49)		
Body mass index			0.06	0.91
<25	231 (64.71)	117 (56.52)		
≥25	126 (35.29)	90 (43.48)		
Previous abdominal operation				
Appendectomy	33 (9.24)	16 (7.73)	0.54	0.07
Cholecystectomy	14 (3.92)	8 (3.86)	0.97	0.68
Hysterectomy	25 (7.00)	14 (6.76)	0.91	0.53
Oophorectomy	9 (2.52)	4 (1.93)	0.65	0.45
Colon-rectal operation	49 (13.73)	11 (5.31)	<0.01	0.41
Total	108 (30.25)	49 (23.67)	0.09	0.98
Comorbidity				
Hypertension	142 (39.78)	92 (44.44)	0.28	0.90
Cardiac disease	26 (7.28)	20 (9.66)	0.32	0.75
Cerebrovascular accident	12 (3.36)	10 (4.83)	0.38	0.99
Asthma	10 (2.80)	6 (2.90)	0.95	0.91
Diabetes mellitus	61 (17.09)	42 (20.29)	0.34	0.73
Liver cirrhosis	3 (0.84)	2 (0.97)	0.88	0.91
Carcinoembryonic antigen (ng/mL)			0.02	0.97
<5	242 (67.79)	159 (76.81)		
≥5	115 (32.21)	48 (23.19)		
Hemoglobin (mg/mL)			0.14	0.98
<10	79 (22.13)	35 (16.91)		
≥10	278 (77.87)	172 (83.09)		
Albumin (mg/dL)			0.03	0.53
<3.5	42 (11.76)	13 (6.28)		
≥3.5	315 (88.24)	194 (93.72)		
Tumor stage			<0.01	0.60
0 and 1	59 (16.53)	63 (30.43)		
2	114 (31.93)	69 (33.33)		
3	122 (34.17)	60 (28.99)		
4	62 (17.37)	15 (7.25)		
T stage			<0.01	0.97
T0	11 (3.08)	10 (4.83)		
T1	17 (4.76)	37 (17.87)		
T2	39 (10.92)	23 (11.11)		
T3	210 (58.82)	111 (53.62)		
T4	80 (22.41)	26 (12.56)		
N stage			0.01	0.79
N0	183 (51.26)	134 (64.73)		
N1	96 (26.89)	44 (21.26)		
N2	78 (21.85)	29 (14.01)		
Histologic type			0.88	0.97
Adenocarcinoma	334 (93.56)	192 (92.75)		
Mucinous adenocarcinoma	4 (1.12)	2 (0.97)		
Signet ring cell	19 (5.32)	13 (6.28)		
Histology grade			<0.01	0.57
Well-differentiated	38 (10.64)	38 (18.36)		
Moderately differentiated	261 (73.11)	158 (76.33)		
Poorly differentiated	5 (1.40)	11 (3.08)		
Retrieved lymph node (+)			0.71	0.91
<12	29 (8.12)	15 (7.25)		
≥12	328 (91.88)	192 (92.75)		
Tumor size (cm)			<0.01	0.67
<4	153 (42.86)	121 (58.45)		
≥4	204 (57.14)	86 (41.55)		

Table 2 shows the surgical data of the groups. Postoperative mortality rate (0.56%, *P*=0.228) and risk of superficial surgical site infection (open vs. laparoscopy, 6.7% vs. 2.4%, *P*<0.001) were significantly higher in the open group than in the laparoscopy group. The overall postoperative morbidity rate (open vs. laparoscopy, 16.5% vs. 9.2%, *P* <0.001) and risks of lung and gastrointestinal complications (lung: open vs. laparoscopy, 1.68% vs. 0.0%, *P*=0.006; gastrointestinal: open vs. laparoscopy, 2.8% vs. 0.97%, *P*=0.026) were also significantly higher in the open group than in the laparoscopy group. One of 207 patients (0.48%) underwent laparoscopic surgery initially and then converted to open surgery because of omentum seeding. The laparoscopy group had a median postoperative hospital stays of 7 days, which was significantly shorter than the 10 days of the open group (*P* < 0.001).

**TABLE 2:** Postoperative Outcomes of Patients Treated With Open Surgery Versus Laparoscopic Surgery, Before and After Propensity Score Weighting

	n (%)		*P*	
	Open Group (N=357)	Laparoscopic Group (N=207)	Before	After
Postoperative mortality	2 (0.56)	0 (0.00)	0.731	0.228
Postoperative morbidity	59 (16.53)	19 (9.18)	0.021	<0.001
Wound (infection, dehiscence, etc.)	24 (6.72)	5 (2.42)	0.042	0.002
Lung (atelectasis, pneumonia, etc.)	6 (1.68)	0 (0.00)	0.147	0.006
Cardiovascular event (MI, CVA, embolism, etc.)	1 (0.28)	1 (0.48)	1.000	0.947
Bladder dysfunction	4 (1.12)	2 (0.97)	1.000	1.000
Gastrointestinal (obstruction, bleeding, etc.)	10 (2.80)	2 (0.97)	0.249	0.027
Abdomen (abscess, peritonitis, etc.)	2 (0.56)	0 (0.00)	0.731	0.390
Anastomosis (leakage, stenosis, etc.)	8 (2.24)	6 (2.90)	0.839	0.287
Others	4 (1.12)	3 (1.45)	1.000	0.620
Conversion	—	1 (0.48)	—	—
Length of hospital stay [medium (range)] (d)	10 (4-63)	7 (3-71)	<0.001	<0.001

CVA indicates cerebrovascular accident; MI, myocardial infarction.

Figure 2 presents the survival of the groups after PSW. The median follow-up times were 48.5 and 24.1 months in the open and laparoscopy groups, respectively. The estimated 5-year OS and DFS were similar between the groups (DFS: open vs. laparoscopy, 72.7% vs. 72.9%; OS: 80.3% vs. 82.0%), and no significant differences were observed in the DFS (Fig. 2A, *P*=0.69) or OS curves (Fig. 2B, *P*=0.85). Table 3 shows the estimated HRs of the groups. PSW revealed that patients receiving laparoscopic surgery had similar prognoses to those receiving open surgery in terms of DFS (PSW-adjusted HR=0.890, 95% confidence interval=0.672-1.179) and OS (PSW-adjusted HR=0.977, 95% confidence interval=0.693-1.377).

**TABLE 3:** Estimated Hazard Ratio of Patients Treated With Open Surgery Versus Laparoscopic Surgery

	Disease-free Survival		Overall Survival	
	Crude HR (95% CI)	PSW-adjusted HR (95% CI)	Crude HR (95% CI)	PSW-adjusted HR (95% CI)
Laparoscopic group	0.687 (0.443-1.065)	0.890 (0.672-1.179)	0.738 (0.428-1.274)	0.977 (0.693-1.377)
Open group	—	—	—	—

CI indicates confidence interval; HR, hazard ratio; PSW, propensity score weighting.

## DISCUSSION

In this study, we analyzed the postoperative short-term and long-term outcomes of patients who underwent laparoscopic radical left hemicolectomy for colon cancer using the standardized 4-step technique at our hospital. Patients in the open surgery group had significantly more advanced TNM stages, poorer nutritional status (albumin), and more abnormal CEA levels than did patients in the laparoscopy group, possibly because of the surgeon’s preferences for patient selection. The systematic differences between the 2 groups were greatly reduced after PSW. Our results showed better short-term outcomes in the laparoscopy group, with lower rates of overall morbidity, superficial surgical site infection, and postoperative pulmonary and gastrointestinal complications as well as shorter hospital stays. Only 0.48% of the laparoscopy group converted to open left hemicolectomy. No significant differences were observed in DFS or OS between the 2 groups. The choice of laparoscopic surgery with the standardized 4-step procedure and FACT method maintained the same long-term outcome than open group.

Previous literature has indicated that compared with anterior resection and right hemicolectomy, left hemicolectomy is associated with longer hospital stay, more complications, and longer operative time. This may indicate that left hemicolectomy including radical dissection of the bilateral midgut and hindgut is technically challenging.^20^ Cases requiring left hemicolectomy account for only 7.5% to 10% of all colon cancers, and 564 (6.98%) of 8075 patients with CRC at CGMH have undergone left hemicolectomy in our 10-year experience^8^,^9^; therefore, further research is needed to understand laparoscopic left hemicolectomy. At our institution, the standardized 4-step technique and FACT method for splenic flexure mobilization in left hemicolectomy were implemented in 2007. Our results showed few postoperative morbidities and short hospital stays in the laparoscopic group for patients with distal transverse colon, splenic flexure, or descending colon cancers after left hemicolectomy; this result is in agreement with previous reports.^11^,^12^,^21^,^22^ The conversion rate was only 0.48% after surgery with our standardized technique, whereas those in other series have ranged from 3.0 to 7.3%.^11^,^12^,^23^–^25^ Moreover, the rate of postoperative surgical complications using our procedure was only 6.28%, which is comparable with those in other studies, ranging from 6% to 33.6%.^23^–^27^ We did not observe any cases of splenectomy associated with iatrogenic splenic injury after using the FACT method for splenic flexure mobilization.

Reports on long-term survival after laparoscopic left hemicolectomy for colon cancer are inconclusive to date. The results of our study showed no difference between the open and laparoscopy groups in long-term oncologic results, including OS and DFS; this finding echoes those of 2 other studies with limited results.^12^,^28^ Although randomized trials on the 4 major colon cancer surgeries have reported the same results—no differences in DFS or OS—3 excluded tumors located in the transverse colon or splenic flexure because such tumors are technically challenging to resect and the related laparoscopic operations are prone to conversion to open surgery.^3^–^7^

The surgical methods in left hemicolectomy differed between surgeons. One meta-analysis showed that the lateral-to-medial approach during laparoscopic colorectal resection might increase procedure time, length of hospital stays, and conversion rate.^29^ Some surgeons introduced different surgical techniques of splenic flexure mobilization with the medial-to-lateral method,^23^,^30^,^31^ but the sample size was limited. In advance, we are the first to show an efficient and safe way to identify the hotspot, entering the lesser sac, and preserving the pancreas from mesocolon securely by the FACT method. Laparoscopic left hemicolectomy following standardized procedures has low complication and conversion rates without compromising oncologic outcomes.

This study emphasizes the importance of standardizing surgical procedures at a training hospital. Several reports have indicated that to reach a steady state, the learning curve for laparoscopic colorectal surgery ranges from 30 to 80 cases.^32^–^35^ A learning curve assessment should consider not only the time required for surgery but also the conversion and complication rates. Because previous studies have shown that complication, conversion, morbidity, and mortality rates decreased as the surgeon’s experience increases,^36^,^37^ we implemented the 4-step technique at CGMH to foster safety, repeatability, and high operative quality for patients. The laparoscopic conversion rate in our study was 0.48%, which is much lower than those of other studies in which the rate has ranged from 7% to 25% in large series and 2% to 41% in smaller series.^38^,^39^ This may be explained by the fact that we trained ourselves in basic surgical skills until we had mastered our 4-step technique to ensure an efficient learning curve. Moreover, the FACT method for splenic flexure mobilization prevents iatrogenic spleen and pancreas injuries that cause excessive bleeding. Another reason could be that the average BMI in Taiwan is lower than that in Europe or the United States, and obesity is reportedly a risk factor for conversion to open surgery.^40^ However, through our standardized surgical training, we have achieved low conversion and postoperative morbidity rates and similar long-term oncologic outcomes to those of open surgery.

This study has several limitations. First, using a retrospective study of a 10-year period might lead to bias between groups. Second, the indications for choice of operative method vary among surgeons, and surgeons played an essential role in the final choice after discussion with patients and their families. Third, the outcomes might be influenced by the technique of the surgeon. Complication rates may be underestimated in the laparoscopy group because surgeons tended to select patients carefully during the early parts of the learning curve. Therefore, this study used the PSW to minimize time-to-event bias between groups and increase statistical power with limited sample size.

## CONCLUSIONS

Our results demonstrate that the standardized 4-step technique and FACT method for splenic flexure mobilization are efficient and safe for performing left hemicolectomy. Laparoscopic left hemicolectomy using such standardized surgical procedures could be applied in treating malignancy.

## Supplementary Material

SUPPLEMENTARY MATERIAL

Supplemental Digital Content is available for this article. Direct URL citations appear in the printed text and are provided in the HTML and PDF versions of this article on the journal's website, www.surgical-laparoscopy.com.

## References

[1] Guillou PJ Quirke P Thorpe H. Short-term endpoints of conventional versus laparoscopic-assisted surgery in patients with colorectal cancer (MRC CLASICC trial): multicentre, randomised controlled trial. Lancet. 2005;365:1718–1726. 15894098 10.1016/S0140-6736(05)66545-2

[2] Veldkamp R Kuhry E Hop WC. Laparoscopic surgery versus open surgery for colon cancer: short-term outcomes of a randomised trial. Lancet Oncol. 2005;6:477–484. 15992696 10.1016/S1470-2045(05)70221-7

[3] Fleshman J Sargent DJ Green E. Laparoscopic colectomy for cancer is not inferior to open surgery based on 5-year data from the COST Study Group Trial. Ann Surg. 2007;246:655–662; discussion 662–664. 17893502 10.1097/SLA.0b013e318155a762

[4] Deijen CL Vasmel JE de Lange-de Klerk ESM. Ten-year outcomes of a randomised trial of laparoscopic versus open surgery for colon cancer. Surg Endosc. 2017;31:2607–2615. 27734203 10.1007/s00464-016-5270-6 PMC5443846

[5] Green BL Marshall HC Collinson F. Long-term follow-up of the Medical Research Council CLASICC trial of conventional versus laparoscopically assisted resection in colorectal cancer. Br J Surg. 2013;100:75–82. 23132548 10.1002/bjs.8945

[6] The Clinical Outcomes of Surgical Therapy Study Group. A comparison of laparoscopically assisted and open colectomy for colon cancer. N Engl J Med. 2004;350:2050–2059. 15141043 10.1056/NEJMoa032651

[7] Colon Cancer Laparoscopic or Open Resection Study Group, Buunen M Veldkamp R. Survival after laparoscopic surgery versus open surgery for colon cancer: long-term outcome of a randomised clinical trial. Lancet Oncol. 2009;10:44–52. 19071061 10.1016/S1470-2045(08)70310-3

[8] COLOR Study Group. COLOR: a randomized clinical trial comparing laparoscopic and open resection for colon cancer. Dig Surg. 2000;17:617–622. 11155008 10.1159/000051971

[9] Kuwabara K Matsuda S Fushimi K. Quantitative comparison of the difficulty of performing laparoscopic colectomy at different tumor locations. World J Surg. 2010;34:133–139. 20020298 10.1007/s00268-009-0292-z

[10] Augier S Ciucci T Luci C. Inflammatory blood monocytes contribute to tumor development and represent a privileged target to improve host immunosurveillance. J Immunol. 2010;185:7165–7173. 21078911 10.4049/jimmunol.0902583

[11] Braga M Frasson M Zuliani W. Randomized clinical trial of laparoscopic versus open left colonic resection. Br J Surg. 2010;97:1180–1186. 20602506 10.1002/bjs.7094

[12] Liang JT Huang KC Lai HS. Oncologic results of laparoscopic versus conventional open surgery for stage II or III left-sided colon cancers: a randomized controlled trial. Ann Surg Oncol. 2007;14:109–117. 17066227 10.1245/s10434-006-9135-4

[13] Kwaan MR Al-Refaie WB Parsons HM. Are right-sided colectomy outcomes different from left-sided colectomy outcomes? Study of patients with colon cancer in the ACS NSQIP database. JAMA Surg. 2013;148:504–510. 23754534 10.1001/jamasurg.2013.1205

[14] Mangano A Gheza F Giulianotti PC. Iatrogenic spleen injury during minimally invasive left colonic flexure mobilization: the quest for evidence-based results. Minerva Chir. 2018;73:512–519. 29658679 10.23736/S0026-4733.18.07737-4

[15] Rosenbaum PR. The central role of the propensity score in observational studies for causal effects. Biometrilca. 1983;70:41–55.

[16] Austin PC. An introduction to propensity score methods for reducing the effects of confounding in observational studies. Multivariate Behav Res. 2011;46:399–424. 21818162 10.1080/00273171.2011.568786 PMC3144483

[17] Austin PC Stuart EA. Moving towards best practice when using inverse probability of treatment weighting (IPTW) using the propensity score to estimate causal treatment effects in observational studies. Stat Med. 2015;34:3661–3679. 26238958 10.1002/sim.6607 PMC4626409

[18] Austin PC. The performance of different propensity score methods for estimating marginal hazard ratios. Stat Med. 2013;32:2837–2849. 23239115 10.1002/sim.5705 PMC3747460

[19] Xie J Liu C. Adjusted Kaplan-Meier estimator and log-rank test with inverse probability of treatment weighting for survival data. Stat Med. 2005;24:3089–3110. 16189810 10.1002/sim.2174

[20] Jamali FR Soweid AM Dimassi H. Evaluating the degree of difficulty of laparoscopic colorectal surgery. Arch Surg. 2008;143:762–767; discussion 768. 18711036 10.1001/archsurg.143.8.762

[21] Han KS Choi GS Park JS. Short-term outcomes of a laparoscopic left hemicolectomy for descending colon cancer: retrospective comparison with an open left hemicolectomy. J Korean Soc Coloproctol. 2010;26:347–353. 21152138 10.3393/jksc.2010.26.5.347 PMC2998025

[22] Lezoche E Feliciotti F Paganini AM. Laparoscopic vs open hemicolectomy for colon cancer. Surg Endosc. 2002;16:596–602. 11972196 10.1007/s00464-001-9053-2

[23] Chenevas-Paule Q Trilling B Sage PY. Laparoscopic segmental left colectomy for splenic flexure carcinoma: a single institution experience. Tech Coloproctol. 2020;24:41–48. 31834555 10.1007/s10151-019-02126-3

[24] de’Angelis N Martinez-Perez A Winter DC. Extended right colectomy, left colectomy, or segmental left colectomy for splenic flexure carcinomas: a European multicenter propensity score matching analysis. Surg Endosc. 2020. [Epub ahead of print]. 10.1007/s00464-020-07431-9 32072288

[25] Ardu M Bergamini C Martellucci J. Colonic splenic flexure carcinoma: is laparoscopic segmental resection a safe enough oncological approach? Surg Endosc. 2019;34:4436–4443. 31617095 10.1007/s00464-019-07221-y

[26] Milone M Angelini P Berardi G. Intracorporeal versus extracorporeal anastomosis after laparoscopic left colectomy for splenic flexure cancer: results from a multi-institutional audit on 181 consecutive patients. Surg Endosc. 2018;32:3467–3473. 29344788 10.1007/s00464-018-6065-8

[27] Kim JC Lee JL Yoon YS. Robotic left colectomy with complete mesocolectomy for splenic flexure and descending colon cancer, compared with a laparoscopic procedure. Int J Med Robot. 2018;14:e1918. 29790253 10.1002/rcs.1918

[28] Desiderio J Trastulli S Ricci F. Laparoscopic versus open left colectomy in patients with sigmoid colon cancer: prospective cohort study with long-term follow-up. Int J Surg. 2014;12:745–750. 24887011 10.1016/j.ijsu.2014.05.074

[29] Hajibandeh S Hajibandeh S Navid A. Meta-analysis of medial-to-lateral versus lateral-to-medial colorectal mobilisation during laparoscopic colorectal surgery. Int J Colorectal Dis. 2019;34:787–799. 30955074 10.1007/s00384-019-03281-7

[30] Okuda J Yamamoto M Tanaka K. Laparoscopic resection of transverse colon cancer at splenic flexure: technical aspects and results. Updates Surg. 2016;68:71–75. 27015933 10.1007/s13304-016-0352-5

[31] Pisani Ceretti A Maroni N Sacchi M. Laparoscopic colonic resection for splenic flexure cancer: our experience. BMC Gastroenterol. 2015;15:76. 26148781 10.1186/s12876-015-0301-7 PMC4494171

[32] Tekkis PP Senagore AJ Delaney CP. Evaluation of the learning curve in laparoscopic colorectal surgery: comparison of right-sided and left-sided resections. Ann Surg. 2005;242:83–91. 15973105 10.1097/01.sla.0000167857.14690.68 PMC1357708

[33] Schlachta CM Mamazza J Seshadri PA. Defining a learning curve for laparoscopic colorectal resections. Dis Colon Rectum. 2001;44:217–222. 11227938 10.1007/BF02234296

[34] Bennett CL Stryker SJ Ferreira MR. The learning curve for laparoscopic colorectal surgery. Preliminary results from a prospective analysis of 1194 laparoscopic-assisted colectomies. Arch Surg. 1997;132:41–44; discussion 45. 9006551 10.1001/archsurg.1997.01430250043009

[35] Dincler S Koller MT Steurer J. Multidimensional analysis of learning curves in laparoscopic sigmoid resection: eight-year results. Dis Colon Rectum. 2003;46:1371–1378; discussion 1378–1379. 14530677 10.1007/s10350-004-6752-5

[36] Marusch FGI Schneider C Scheidbach H. Laparoscopic Colorectal Surgery Study Group (LCSSG). Experience as a factor influencing the indications for laparoscopic colorectal surgery and the results. Surg Endosc. 2001;15:116–120. 11285950 10.1007/s004640000340

[37] Larach SWPS Ferrara A Williamson PR. Complications of laparoscopic colorectal surgery: analysis and comparison of early vs. later experience. Dis Colon Rectum. 1997;40:592–596. 9152190 10.1007/BF02055385

[38] Marusch FGI Schneider C Scheidbach H. Laparoscopic Colorectal Surgery Study Group (LCSSG). Importance of conversion for results obtained with laparoscopic colorectal surgery. Dis Colon Rectum. 2001;44:207–214. 11227937 10.1007/BF02234294

[39] Gervaz PPA Utech M Secic M. Converted laparoscopic colorectal surgery. Surg Endosc. 2001;15:827–832. 11443444 10.1007/s004640080062

[40] Bell S Kong JC Wale R. The effect of increasing body mass index on laparoscopic surgery for colon and rectal cancer. Colorectal Dis. 2018;20:778–788. 29577556 10.1111/codi.14107

